# *I-f* Control Method for SynRMs Uses Simple Inductance Identification and Voltage Injection for Current and Angle Control

**DOI:** 10.3390/s24247970

**Published:** 2024-12-13

**Authors:** Yibo Guo, Lingyun Pan, Yang Yang, Yimin Gong, Xiaolei Che

**Affiliations:** College of Physics, Jilin University, Changchun 130000, China; guoyb22@mails.jlu.edu.cn (Y.G.); ply@jlu.edu.cn (L.P.); gongym@jlu.edu.cn (Y.G.); chexl@jlu.edu.cn (X.C.)

**Keywords:** current frequency (*I–f*) control, synchronous reluctance motor (SynRM), startup strategy, voltage injection

## Abstract

The sensorless vector control method of synchronous reluctance motors (SynRMs), based on extended back electromotive force (EMF) or flux observation, has been widely applied in the medium- or high-speed range. However, in the low-speed and low-current range, the extended back-EMF and flux are nearly zero. The use of the current frequency (*I-f*) control method can enable the motor to pass through the low-speed region, thereby ensuring that the back-EMF and flux reach a large value. *I-f* control methods that are widely used in permanent magnet synchronous motors (PMSMs) may encounter many problems when applied to SynRMs. The most serious issue is the inability to adjust the current amplitude to control the rotor angle and achieve a smooth transition to sensorless control. Based on various issues, this article proposes an *I-f* control method with four stages that can be used in SynRMs. This method uses a simple inductance identification method to solve the flux saturation phenomenon of SynRMs and then uses high-frequency voltage injection to continuously adjust the current amplitude and rotor angle position in conjunction with this inductance identification method. The effectiveness of this method is experimentally demonstrated on a 5.5 kW SynRM.

## 1. Introduction

In recent years, the synchronous reluctance motor (SynRM), as a type of motor that does not use permanent magnets, has shown strong competitiveness compared to the permanent magnet synchronous motor (PMSM) in cost-sensitive application scenarios. Compared to induction motors (IMs), synchronous reluctance motors do not have windings on the rotor, which makes their structure simple, sturdy, and more efficient. In addition, synchronous reluctance motors also have advantages such as resistance to harsh environments, low maintenance costs, and a wide speed range. In order to fully utilize these advantages, it is necessary to achieve speed sensorless control. Methods used in motors to achieve sensorless control include using a Kalman Filter [1,2], extended back electromotive force (EMF) [3,4], flux observers [5,6], high-frequency signal injection [7,8], etc. These methods can estimate the rotor position and speed.

Among these different control methods, the methods using a Kalman Filter have not been widely applied due to their huge computational complexity. The methods of injecting high-frequency signals include voltage injection [7] and current injection [8]. The voltage injection method requires the addition of voltage vectors in the subsequent field-oriented control (FOC). In high-power application scenarios, due to the maximum limit of voltage vector values, it may cause distortion; this will reduce the signal-to-noise ratio of the induced signal, which can affect motor control. However, the current injection method requires the consideration of hardware and software limitations on the current, and the effective value may be forced to decrease, which may prevent the torque from reaching its maximum. Based on the drawbacks of the above methods, in the high-speed operating range of motors, the methods of using extended back-EMF and flux observation to achieve rotor position and speed estimation are more popular. However, these methods perform poorly in the low-speed and low-current operating range because the extended back-EMF and flux are close to zero. Therefore, some methods need to be used to quickly start the motor at a certain speed to get through the low-speed range. The high-frequency signal injection method can be used to overcome the low-speed operation stage of the motor, but due to the need to consider issues such as observer convergence speed and loop stability, the current regulation speed is not very fast, which results in a generally long time for the motor speed to climb. Besides this method, the voltage frequency (*V-f*) control method [9,10,11,12] and current frequency (*I-f*) control method [13,14,15,16,17,18,19,20,21,22,23,24,25] are often chosen to solve this problem.

Among these two methods, the *V-f* control method has a major problem in that it cannot control the stator current, making it prone to oscillation. In addition, the requirement of compensating for stator resistance voltage drop and inverter voltage error will also increase the difficulty of smoothly implementing the *V-f* control method. When switching from *V-f* to closed-loop sensorless control, there is also a certain difficulty in transmitting the state of the motor. In the *I-f* control method, the current is regulated through closed-loop control and the motor current and voltage can be easily obtained from the current regulator, making it easier to transmit the current and voltage states to the subsequent closed-loop sensorless control, thereby achieving smoother transition. This means that the *I-f* control method is more suitable as a startup strategy for a motor.

The *I-f* control method has been continuously improved to solve many problems, and existing methods have been proven effective on PMSMs [13,14,15,16,17,18,19,20,21,22]. However, these methods have many unreasonable aspects when directly applied to SynRMs. There are some studies on the fast startup strategy of SynRMs [12,23,24,25], but these studies all have some issues. The proposed method in [12] uses *V-f* control, but based on the above drawbacks of *V-f* control, it is not considered to be used. In [23,24,25], the design of the current regulator is unreasonable because the mathematical model of SynRMs has not been properly analyzed, and the voltage output by a current regulator should not be obtained solely from its axial current error. The method in [24] directly ignores the *q*-axis inductance; this method has the potential for improvement. The control strategy in [25] sets the *q*-axis current to zero, which is unreasonable. In PMSMs, the *d*-axis current is often set to zero, which makes control more convenient [17,18,19]. However, for SynRMs, the torque will be zero when the *q*-axis or *d*-axis current is zero. Reducing the current amplitude and adjusting the rotor angle position are key to switching from *I-f* control to sensorless control, and all existing methods cannot be implemented smoothly on an SymRM.

Based on the lack of a reasonable *I-f* control method for SynRMs, and unlike PMSMs, SynRMs exhibit flux saturation, the *d-q* axis current cannot be zero. This article proposes a new *I-f* control method. This method has four stages, namely the speed-up stage, current-down stage, angle adjustment state, and sensorless control state. This method combines a simple inductance identification method to avoid complex motor model identification [26,27], continuously reduces current amplitude, and determines the rotor position by injecting high-frequency voltage, thereby achieving smooth switching to closed-loop sensorless control. At the same time, this method uses the strategy of f∝t, as used in [20], and also combines power compensation for frequency correction to reduce speed oscillations, which is used in [17,18,19].

The main contribution of this article is to propose an *I-f* control method that can be used on SynRMs, which is introduced in Section 4. When it is determined that the motor is operating in the high-speed range, this method can be used to quickly cross the low-speed range of the motor. This article also provides a detailed analysis of the mathematical model of an SynRM and explains why existing methods applicable to PMSMs cannot be used for SynRMs, which are shown in Section 2 and Section 3.

The remainder of this article is organized as follows. The analysis of SynRMs models with *I-f* control is presented in Section 2. In Section 3, detailed analysis is conducted on the issues when traditional *I-f* control methods are applied to SynRMs. The principles and implementation methods of the *I-f* control method proposed for these issues are described in Section 4. The effectiveness of the *I-f* control method was experimentally confirmed, and the results are shown in Section 5. The experimental results of using the high-frequency voltage injection method with the same motor for low-speed starting can be found in [28], and the results were also compared during the experimental process. Finally, Section 6 concludes this article.

## 2. Mathematical SynRM Model with *I-f* Control

### 2.1. The Mathematical Model of an SynRM

The model of an SynRM in a *d-q* axis synchronous frame under steady state can be expressed as

(1) $$\left[\begin{array}{c} u_{d}\\ u_{q} \end{array}\right]=\left[\begin{array}{cc} R_{s} & -\omega_{e}L_{q}\\ \omega_{e}L_{d} & R_{s} \end{array}\right]\left[\begin{array}{c} i_{d}\\ i_{q} \end{array}\right]+\left[\begin{array}{cc} L_{d} & 0\\ 0 & L_{q} \end{array}\right]\left[\begin{array}{c} \frac{di_{d}}{dt}\\ \frac{di_{q}}{dt} \end{array}\right]$$

where $u_{d}$, $u_{q}$, $R_{s}$, $i_{d}$, $i_{q}$, $\omega_{e}$, $L_{d}$, and $L_{q}$ are the *d*-axis stator voltage, *q*-axis stator voltage, stator resistance, *d*-axis stator current, *q*-axis stator current, motor electrical angular speed, *d*-axis inductance, and *q*-axis inductance.

The electromagnetic torque mathematic model and the motor motion equation can be expressed as

(2) $$T_{e}=\frac{3P}{2}\left(L_{d}-L_{q}\right)i_{d}i_{q}$$

(3) $$J\frac{d\omega_{r}}{dt}=T_{e}-T_{L}-B\omega_{r}$$

where *P* is the number of pole pairs; J is the rotor inertia; $T_{L}$ is the load torque; $\omega_{r}$ is motor speed; where $\omega_{r}=\omega_{e}/P$; and *B* is the damping coefficient.

### 2.2. Dynamic Analysis of SynRM with I-f Control Method

**Figure 1:**
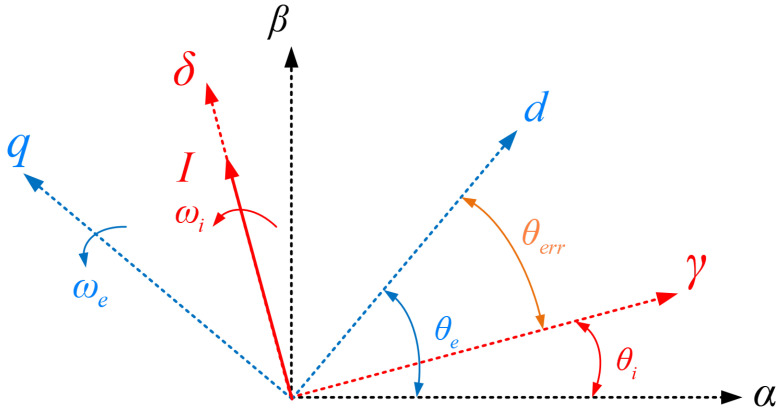
Schematic of *d-q* and *γ-δ* coordinate system.

Regarding the *I-f* control method, the actual rotor position is generally unknown, and a new coordinate axis system *γ-δ* is defined to represent the direction of current, as shown in Figure 1. The current direction is in the *δ* direction, the electrical angular speed of the current is $\omega_{i}$, the electrical angle of the *γ-δ* coordinate system is $\theta_{i}$, the actual electrical angle of the rotor is $\theta_{e}$, the electrical angular speed of the rotor is $\omega_{e}$, and the angle between the two coordinate systems is *θ_err_*, which can be expressed as

(4) $$\theta_{err}=\theta_{e}-\theta_{i}$$

When the current amplitude *I* is determined, the electromagnetic torque $T_{e}$ of the motor can be calculated using (2), which can be expressed as

(5) $$T_{e}=\frac{3P}{4}\left(L_{d}-L_{q}\right)I^{2}sin2\theta_{err}$$

**Figure 2:**
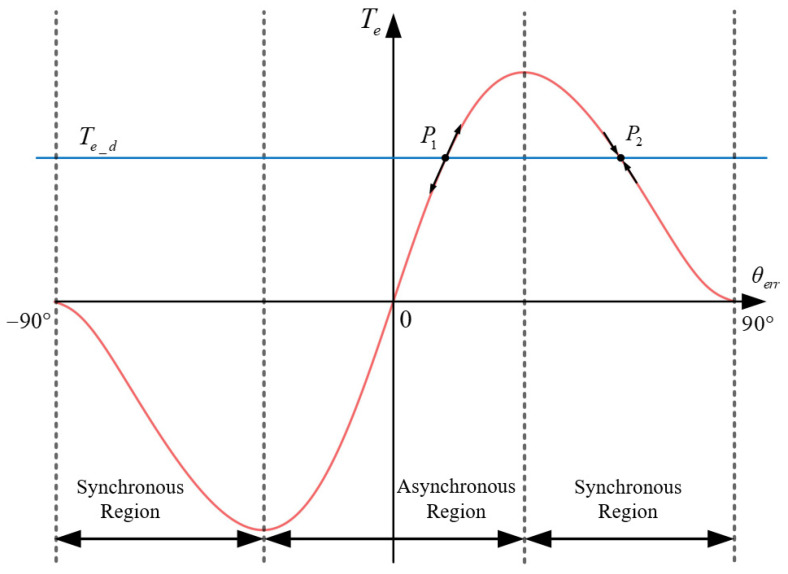
Schematic of the variation in torque generated with angle difference *θ_err_*.

According to (3), the demand torque $T_{e\_d}$ consists of the load torque $T_{L}$, the acceleration torque $J\frac{d\omega_{r}}{dt}$, and the damping torque $B\omega_{r}$. When the demand torque $T_{e\_d}$ is constant, combining electromagnetic torque expression (5) can obtain the results in Figure 2. As shown in the figure, there are two intersections, *P*_1_ and *P*_2_. But for the stability of point *P*_1_, it can be seen that *P*_1_ can be considered a source point, and the angle difference *θ_err_* cannot remain stable at this point. And *P*_2_ can be considered a sink point, where the angle difference *θ_err_* can remain stable. Therefore, the operation region of the current can be divided into two types, a synchronous region and asynchronous region. When the SynRM is well controlled using the *I-f* method, the operating point will always be within the synchronous region.

**Figure 3:**
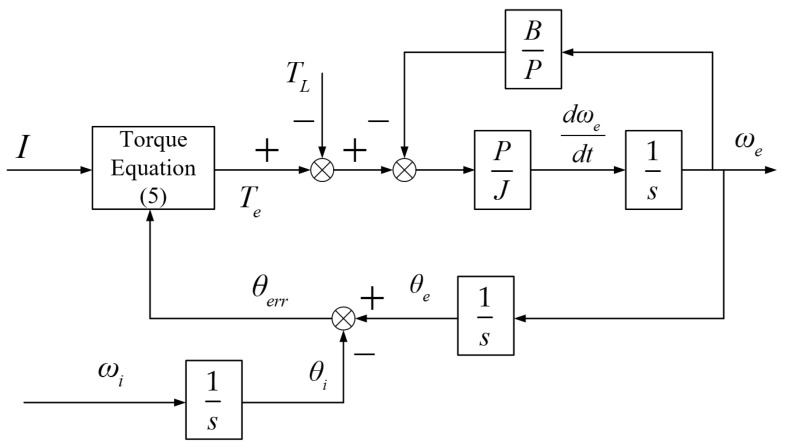
Large-signal motion model of SynRM under *I-f* control.

A system diagram of the motor is shown in Figure 3, where the amplitude of the current *I* and the electrical angular speed of the current $\omega_{i}$ are used as system inputs, and the electrical angular speed of the rotor $\omega_{e}$ is used as the system output. This is commonly referred to as the large-signal motion model of an SynRM under *I-f* control.

### 2.3. The Mathematical Model of an SynRM Within a γ-δ Coordinate System

Due to the difference between SynRMs and PMSMs, and the fact that the electromagnetic torque of an SynRM is mainly reluctance torque, its maximum value does not exist when $\theta_{err}=0$, and simplified calculations cannot be used. The model of an SynRM in the *γ-δ* axis synchronous frame can be obtained using a linearity transformation matrix, which is

(6) $$R=\left[\begin{array}{cc} cos\theta_{err} & -sin\theta_{err}\\ sin\theta_{err} & cos\theta_{err} \end{array}\right]$$

This linear transformation matrix can convert the system’s state variables between coordinate axes:

(7) $$\left[\begin{array}{c} u_{\gamma }\\ u_{\delta } \end{array}\right]=R\left[\begin{array}{c} u_{d}\\ u_{q} \end{array}\right],\;\left[\begin{array}{c} i_{\gamma }\\ i_{\delta } \end{array}\right]=R\left[\begin{array}{c} i_{d}\\ i_{q} \end{array}\right]$$

It can be obtained from Figure 1 and Equation (4) that

(8) $$\frac{d\theta_{err}}{dt}=\omega_{e}-\omega_{i}$$

In combining (1), (6), (7), and (8), the expression of an SynRM in the *γ-δ* coordinate system can be obtained:

(9) $$\left[\begin{array}{c} u_{\gamma }\\ u_{\delta } \end{array}\right]=\left[\begin{array}{cc} A_{11} & A_{12}\\ A_{21} & A_{22} \end{array}\right]\left[\begin{array}{c} i_{\gamma }\\ i_{\delta } \end{array}\right]+\left[\begin{array}{cc} B_{11} & B_{12}\\ B_{21} & B_{22} \end{array}\right]\left[\begin{array}{c} \frac{di_{\gamma }}{dt}\\ \frac{di_{\delta }}{dt} \end{array}\right]$$

where

(10) $$\left\{\begin{array}{c} B_{11}=L_{d}cos^{2}\theta_{err}+L_{q}sin^{2}\theta_{err}\\ B_{12}=B_{21}=\frac{1}{2}\left(L_{d}-L_{q}\right)sin2\theta_{err}\\ B_{22}=L_{d}sin^{2}\theta_{err}+L_{q}cos^{2}\theta_{err}\\ A_{11}=R_{s}-B_{12}\left(2\omega_{e}-\omega_{i}\right)\\ A_{12}=\left(L_{d}-L_{q}\right)\omega_{e}cos2\theta_{err}-B_{11}\omega_{i}\\ A_{21}=\left(L_{d}-L_{q}\right)\omega_{e}cos2\theta_{err}+B_{22}\omega_{i}\\ A_{22}=R_{s}+B_{21}\left(2\omega_{e}-\omega_{i}\right) \end{array}\right.$$

The use of *I-f* control for SynRMs should be designed and analyzed using (9) and (10).

## 3. The Limitations of Traditional *I-f* Control Methods on SynRMs

### 3.1. Overview of Existing I-f Control Methods

**Figure 4:**
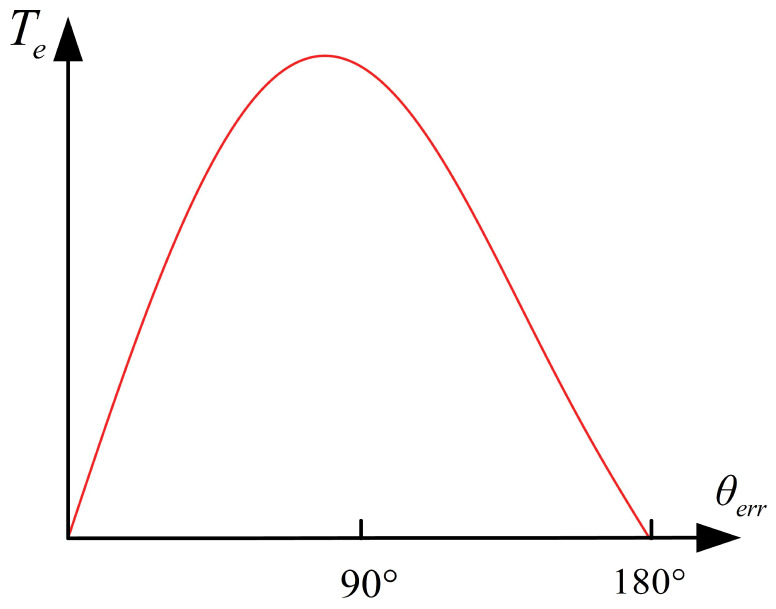
Schematic of the variation in torque generated with angle difference *θ_err_* on PMSMs.

This section will demonstrate the limitations of applying *I-f* control methods applicable to PMSMs to SynRMs. According to the analysis in Section 2.2, the *I-f* control method can achieve high motor torque, which involves adapting different operating conditions using a larger current amplitude. But larger currents can lead to low efficiency. For PMSMs, a schematic of the variation in torque generated with angle difference *θ_err_* is shown in Figure 4. It can be clearly seen that when $\theta_{err}=\pi /2$, the torque can be approximated as the maximum; at this point, for a certain $T_{e,d}$, the current amplitude is at its minimum. For SynRMs, however, when $\theta_{err}=\pi /4$, the current amplitude *I* can reach its minimum value, which can effectively improve efficiency. A more serious issue is that the specific rotor position is unknown, which makes it difficult to switch to sensorless control methods. Forceful switching will cause a sudden change in the current and voltage vector. This leads to problems in current control or makes the torque operation point into the asynchronous region, resulting in motor loss synchronization. In order to solve the above two problems, studies on *I-f* control methods have focused on current amplitude and angle control, but existing methods cannot be used on SynRMs. This section will analyze in detail the reasons why the existing methods cannot be used.

### 3.2. Flux Saturation Phenomenon of SynRMs

**Figure 5:**
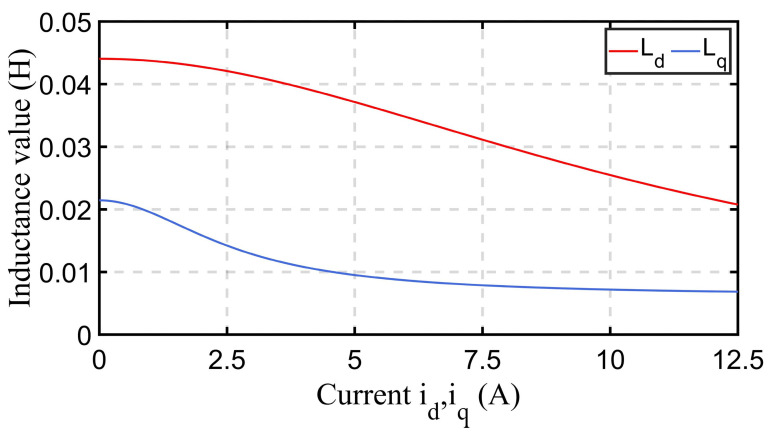
Schematic of *d-q* axis inductance changing with axial current.

Previously, the mathematical model of an SynRM was presented to further analyze the application of *I-f* control methods. However, SynRMs exhibit the flux saturation phenomenon, which causes the inductance of its *d-q* axis to change along with the magnitude of the axial current. A schematic of inductance variation is shown in Figure 5. Obtaining *d-q* axis inductance at different current amplitudes has become necessary preparatory work. However, some existing methods [26,27] are too complex and require a large amount of data to be processed.

### 3.3. Limitations of Existing Methods for Reducing Current Amplitude and Angle Control

The existing *I-f* control has been widely applied to PMSMs. In order to improve efficiency and avoid sudden changes in current and voltage vectors when switching to closed-loop sensorless control, angle control is usually performed by adjusting the angle *θ_err_* between the *d-q* axis and the *γ-δ* axis.

As described in [18], the motor torque equation in the large-signal motion model is nonlinear, making it particularly difficult to analyze and design controllers. A method was proposed in [18]; in extracting a small-signal motion model form the large-signal motion model, which first linearized the torque, for PMSMs, it can be expressed as

(11) $$T_{e}=\frac{3P}{2}\left[\left(L_{d}-L_{q}\right)i_{d}+\lambda_{f}\right]i_{q}=\frac{3P}{2}\left[\left(L_{d}-L_{q}\right)Isin\theta_{err}+\lambda_{f}\right]Icos\theta_{err}$$

In taking the derivative of the above equation, it can be obtained that

(12) $$\left\{\begin{array}{c} \Delta T_{e}=K_{I}\Delta I-K_{\theta }\Delta \theta_{err}\\ K_{I}=\frac{\partial T_{e}}{\partial I}=\frac{3P}{2}\left[\left(L_{d}-L_{q}\right)Isin2\theta_{err}+\lambda_{f}cos\theta_{err}\right]\\ K_{\theta }=\frac{\partial T_{e}}{\partial \theta_{err}}=\frac{3P}{2}\left[\left(L_{d}-L_{q}\right)I^{2}cos2\theta_{err}+\lambda_{f}cos\theta_{err}\right] \end{array}\right.$$

where $\lambda_{f}$ is the permanent magnet flux linkage, and $K_{I}$ and $K_{\theta }$ is the small-signal gains. With these two gains, a current amplitude controller and an angle controller can be further analyzed and designed, which can gradually reduce the current amplitude and achieve the desired angle value.

For SynRMs, the torque equation is shown in (5), and the two gains can be written with the condition $\theta_{err}=\pi /4$ as

(13) $$\left\{\begin{array}{c} K_{I}=\frac{3P}{2}\left(L_{d}-L_{q}\right)I\\ K_{\theta }=0 \end{array}\right.$$

Obviously, it is impossible to further design controllers to control the current amplitude and angle based on these two gains; the idea of linearizing torque is no longer applicable.

### 3.4. Design Issues of Current Regulators

The essence of the *I-f* control method is to use a rotating current to quickly start the motor, and the accuracy of the current is crucial. Therefore, the current regulator is very important in the *I-f* control method. It must have characteristics such as precise control and fast response. Proportional–integral (PI) regulators possess the aforementioned advantages. For PMSMs, as shown in Figure 4, when $\theta_{err}=\pi /2$, the direction of the current injection coincides with the *q*-axis, and ignoring iron loss makes it easy to design a PI regulator with its own axial direction. But for SynRMs, their voltage expression shows a very obvious coupling phenomenon on the *γ-δ* axis, which is shown in (9) and (10); traditional PI regulators cannot be used in this situation.

## 4. Proposed *I-f* Control Methods on SynRMs

Based on the flux saturation phenomenon of SynRMs, it is necessary to calibrate the inductance in order to control the motor. A very simple method for inductance calibration has been proposed, and the subsequently proposed *I-f* control method can achieve reducing current amplitude and rotor position estimation based on this identification method, thereby solving the problem that existing *I-f* control methods cannot be implemented on SynRMs.

### 4.1. A Simple Inductance Identification Method for SynRMs

A high-frequency voltage expression can be designed as follows:

(14) $$u_{ref}\left(k\right)=\left\{\begin{array}{c} u_{inj}+R_{s}i\left(k\right),\mathrm{if}\;i\left(k\right)<I_{switch}-I_{border}\\ -u_{inj}+R_{s}i\left(k\right),\mathrm{if}\;i\left(k\right)>I_{switch}+I_{border}\\ u_{ref}\left(k-1\right),\mathrm{otherwise} \end{array}\right.$$

If this voltage is applied to an inductor with a resistance of $R_{s}$, it can be seen that the current value of the inductor can be approximated as repeatedly rise and fall within the range of $\left[I_{switch}-I_{border},I_{switch}+I_{border}\right]$. Record the maximum current value $I_{h}$ and minimum current value $I_{l}$ during a certain voltage direction switch process. When $I_{border}$ is a small value, it can be considered that the inductance value of the inductor is

(15) $$L\left(I_{switch}\right)=\frac{u_{inj}\Delta T}{I_{h}-I_{l}}$$

where ΔT is the duration of this voltage switch process. (15) can be considered the inductance value of the inductor when its current value is $I_{switch}$. $I_{border}$ is a fixed value; when the rated current of the motor is $I_{max}$, $I_{switch}$ is expressed as

(16) $$I_{switch}=\frac{nI_{max}}{\sqrt{2}m}\;\left(n=m,m-1,\ldots ,1\right)$$

where m is a manually defined positive integer, whenever there are several voltage switching processes, n decreases by 1.

**Figure 6:**
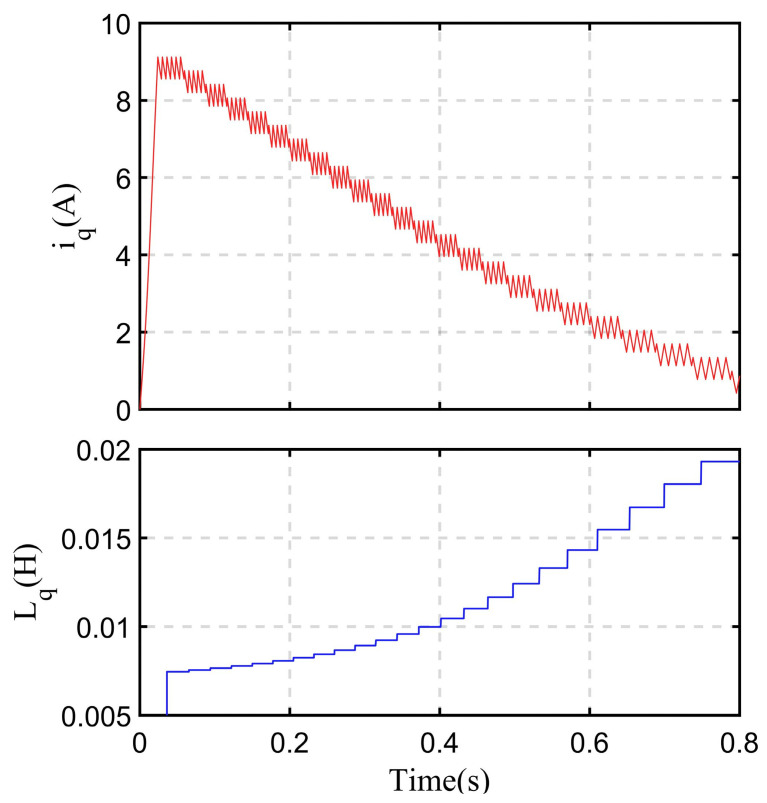
Schematic of identifying the *q*-axis inductance.

Applying the above analysis to SynRMs can obtain their inductance values at different current values. Firstly, it is necessary to locate the rotor angle. From Figure 1 and Figure 2, it can be seen that when injecting current in a certain direction, for a motor with no load, its rotor will rotate to the *d*-axis in the same direction as the injected current. Therefore, current can be injected into A-phase to ensure that the motor rotor is in the $\theta_{e}=0$ state at the beginning of the experiment. Afterward, with the combination of (14)–(16), inject voltage into the *d-q* axis in sequence, record the current and time, and calculate the magnitude of the motor axial inductance at different current values. Figure 6 is a schematic of calibrating the *q*-axis inductance, with $I_{max}=12.5A$ and m = 25, where n decreases by 1 every ten times the voltage direction is switched.

### 4.2. Detecting Angle Difference θ_err_ Using Injection Voltage

From (9) and (10), it can be seen that when the motor is in operation with well-controlled current, injecting high-frequency square-wave-type voltage into the *δ*-axis will generate high-frequency induced current in the *γ-δ* axis. This induced current will not affect the effective value of the current, so only the differential part can be considered to calculate the induced current. The square-wave-type voltage is represented as

(17) $$V_{inj}\left(t\right)=\left\{\begin{array}{c} V\;\;\;\;\;\;\;(2NT\leq t<2NT+T)\\ -V\;\;\;(2NT+T\leq t<2NT+2T) \end{array}\right.$$

where *V* is the amplitude of the injected voltage, which is a positive value; *N* is a positive integer; and *T* is half of the voltage period, which is a very small time. Calculate the induced current based on the positive half-cycle of the injected voltage.

(18) $$\left[\begin{array}{c} 0\\ VT \end{array}\right]=\left[\begin{array}{cc} B_{11} & B_{12}\\ B_{21} & B_{22} \end{array}\right]\left[\begin{array}{c} \Delta i_{\gamma }\\ \Delta i_{\delta } \end{array}\right]$$

where Δ*i_γ_* and Δ*i_δ_* are variations in *i_γ_* and *i_δ_* during this half-voltage injection cycle. And the most critical factor in determining angle difference *θ_err_* is the Δ*i_γ_*, which can be solved using (10).

(19) $$\Delta i_{\gamma }=\frac{\left(L_{d}-L_{q}\right)VTsin2\theta_{err}}{2L_{d}L_{q}}$$

From this expression, it can be seen that when $\theta_{err}=\pi /4$, the current change Δ*i_γ_* reaches its maximum and is $\frac{\left(L_{d}-L_{q}\right)VT}{2L_{d}L_{q}}$. The previously proposed identification method for inductance can calibrate the axial inductance when the *I-f* control current amplitude is $\frac{nI_{max}}{m}$ and the angle difference $\theta_{err}=\pi /4$. Therefore, the current variable here can be accurately calculated. So, this method can accurately determine the situation where $\theta_{err}=\pi /4$.

### 4.3. Proposed I-f Control Method with Four Stages

**Figure 7:**
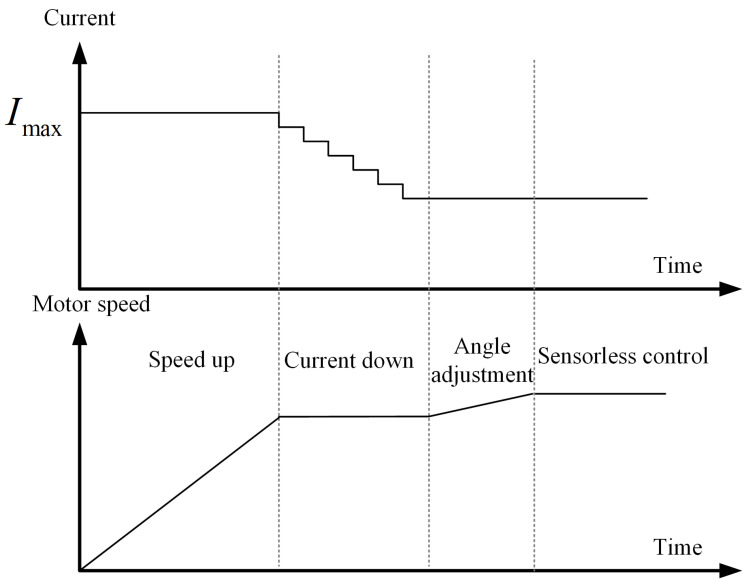
Current amplitude and speed of the proposed *I-f* control method with four states.

The proposed *I-f* control method has four stages, and a schematic of its current amplitude and speed is shown in Figure 7. The four stages are the speed-up state, current-down state, angle adjustment state, and sensorless control state.

**Figure 8:**
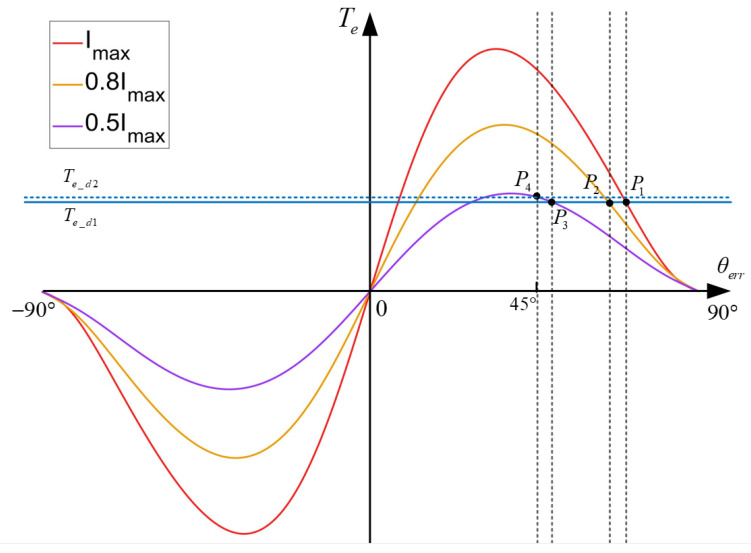
The variation in motor operating status on torque image.

In the speed-up state, the rated current of the motor $I_{max}$ is used as the injection current amplitude, and the motor speed slowly increases at a fixed slope to 5% of the rated speed of the motor. In the current-down state, the motor speed remains unchanged, and the current will decrease in a stepwise manner with $\frac{I_{max}}{m}$ as the step size. Using a fixed step size to reduce the current amplitude can ensure the accuracy of the identified inductance used. In the angle adjustment state, slowly increase the speed of the motor, which means constantly raising the injection frequency of the current, so that *θ_err_* gradually reaches π/4. When $\theta_{err}=\pi /4$, immediately switch to sensorless control. The performance of the entire process on the torque image is shown in Figure 8. At the end of the speed-up state, angle difference *θ_err_* stabilizes at *P*_1_ and then gradually moves to *P*_2_ in the current-down stage, reaching *P*_3_ at the end of this state. In the angle adjustment state, the motor speed is slowly increased to increase some damping torque, and the final correction of the angle difference $\theta_{err}=\pi /4$. At this point *P*_4_, the motor control will switch to the sensorless control state.

As described above, when the proposed *I-f* control is applied to SynRMs, its time duration of the method and each state are random, and the final achieved speed is unknown. However, the use of this method can ensure that when the motor parameters change, due to the existence of inductance identification methods, suitable motor parameters for (19) can be accurately selected, thereby achieving smooth state transition and accurate current position control.

The method of using voltage injection to detect angle difference *θ_err_* introduced earlier will be applied during the current-down state and angle adjustment state, and the switching between the two stages is also judged with the assistance of this method. According to torque expression (5), it can be seen that when the current amplitude changes from $\frac{nI_{max}}{m}$ to $\frac{\left(n-1\right)I_{max}}{m}$ and *θ_err_* remains unchanged, the torque will change to its original $\frac{{\left(n-1\right)}^{2}}{n^{2}}$. *I-f* control has the ability to self-adjust, and these changes will be compensated for by changes in *θ_err_*. *θ_err_* will change according to the following expression:

(20) $$sin2\theta_{err,n-1}=\frac{n^{2}}{{\left(n-1\right)}^{2}}sin2\theta_{err,n}$$

In order to prevent excessive *θ_err_* changes from entering the asynchronous region, the decrease in current needs to be stopped in a timely manner. In combining (19) and (20), it can be concluded that when the value of Δ*i_γ_* is detected as $\frac{{\left(n-1\right)}^{2}\left(L_{d}-L_{q}\right)VT}{2n^{2}L_{d}L_{q}}$, the decrease in current should be stopped, as another decrease would result in $\theta_{err}<\pi /4$. The motor cannot provide the required electromagnetic torque, and the motor operation point enters the asynchronous region.

### 4.4. Current Regulator of Proposed I-f Control Method

The mathematical model of SynRMs in the *δ-γ* axis exhibits severe coupling phenomena, and if a simple PI is used, it cannot effectively regulate the current, which will fundamentally affect the usability of the *I-f* control method.

For the convenience of designing current regulators, when the *I-f* control method proposed in this article operates reasonably, it can be approximately assumed that $\theta_{err}=\pi /4$ and $\omega_{e}=\omega_{i}$. Substitute these into (10) and define

(21) $$\left\{\begin{array}{c} A=\left[\begin{array}{cc} R_{s}-\frac{1}{2}\omega_{i}\left(L_{d}-L_{q}\right) & -\frac{1}{2}\omega_{i}\left(L_{d}+L_{q}\right)\\ \frac{1}{2}\omega_{i}\left(L_{d}+L_{q}\right) & R_{s}-\frac{1}{2}\omega_{i}\left(L_{d}-L_{q}\right) \end{array}\right]\\ B=\frac{1}{2}\left[\begin{array}{cc} L_{d}+L_{q} & L_{d}-L_{q}\\ L_{d}-L_{q} & L_{d}+L_{q} \end{array}\right]\\ \begin{array}{l} \;X=\left[\begin{array}{c} i_{\gamma }\\ i_{\delta } \end{array}\right]\\ X^{*}=\left[\begin{array}{c} {i_{\gamma }}^{*}\\ {i_{\delta }}^{*} \end{array}\right] \end{array} \end{array}\right.$$

The target value is marked with an * symbol. The current error between the true value and the target value is $e=X^{*}-X$. Set the system input *U* as an integrator expressed in the form of

(22) $$U=\int \frac{2\omega_{c}}{T_{s}}Be\mathrm{dt}$$

where $T_{s}$ is the control cycle time. In discretizing the error system with this time length, the expression can be obtained by combining (9), (10), (21), and (22):

(23) $$\dot{e}=-B^{-1}Ae-2B^{-1}\omega_{c}Be$$

After calculating and organizing, it can be obtained that

(24) $$\dot{e}=-\frac{1}{2}\left[\begin{array}{cc} R_{s}\left(\frac{1}{L_{d}}+\frac{1}{L_{q}}\right)+4\omega_{c} & R_{s}\left(\frac{1}{L_{d}}-\frac{1}{L_{q}}\right)+2\omega_{i}\\ R_{s}\left(\frac{1}{L_{d}}-\frac{1}{L_{q}}\right)+2\omega_{i} & R_{s}\left(\frac{1}{L_{d}}+\frac{1}{L_{q}}\right)+4\omega_{c} \end{array}\right]e$$

This is a system of first-order linear equations. The two eigenvalues $\lambda_{1}$, $\lambda_{2}$ of the above matrix satisfy

(25) $$\left\{\begin{array}{c} \lambda_{1}+\lambda_{2}=-R_{s}\left(\frac{1}{L_{d}}+\frac{1}{L_{q}}\right)-4\omega_{c}\\ \lambda_{1}\lambda_{2}={\left(R_{s}\left(\frac{1}{L_{d}}+\frac{1}{L_{q}}\right)+4\omega_{c}\right)}^{2}-{\left(R_{s}\left(\frac{1}{L_{d}}-\frac{1}{L_{q}}\right)+2\omega_{i}\right)}^{2} \end{array}\right.$$

If $\omega_{c}>0.5\omega_{i}$, then $\lambda_{1}+\lambda_{2}<0$ and $\lambda_{1}\lambda_{2}>0$. That is, $\lambda_{1}<0$ and $\lambda_{2}<0$. The above error system converges to zero, and *X* will be equal to $X^{*}$. To prove that (22) is feasible as a current regulator, divided into two parts, it can be expressed as

(26) $$\left\{\begin{array}{c} u_{\gamma }=\int \frac{\omega_{c}}{T_{s}}\left(L_{d}+L_{q}\right)\left({i_{\gamma }}^{*}-i_{\gamma }\right)dt+\int \frac{\omega_{c}}{T_{s}}\left(L_{d}-L_{q}\right)\left({i_{\delta }}^{*}-i_{\delta }\right)dt\\ u_{\delta }=\int \frac{\omega_{c}}{T_{s}}\left(L_{d}+L_{q}\right)\left({i_{\delta }}^{*}-i_{\delta }\right)dt+\int \frac{\omega_{c}}{T_{s}}\left(L_{d}-L_{q}\right)\left({i_{\gamma }}^{*}-i_{\gamma }\right)dt \end{array}\right.$$

To make the system converge quickly, a proportional term can be added to form a proportional–integral (PI) regulator. This will not affect the stability of the system.

### 4.5. Using the Proposed I-f Control Method for Fast SynRM Startup

**Figure 9:**
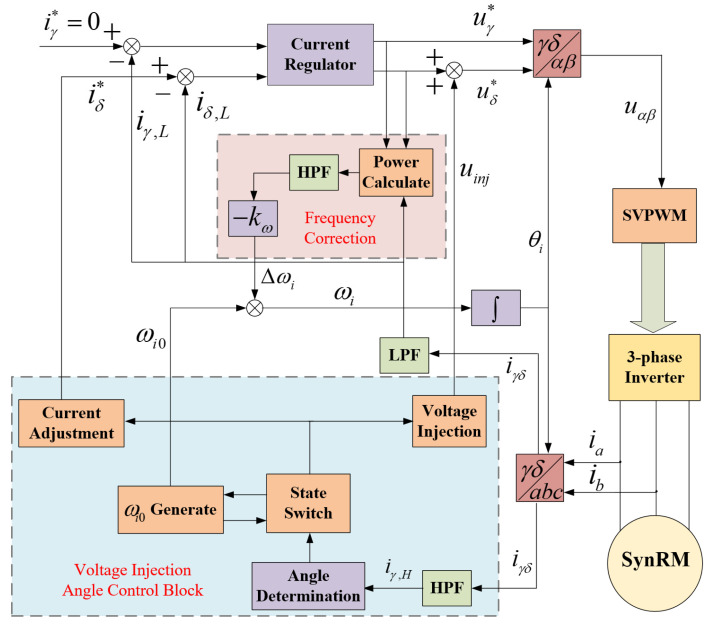
Schematic of the proposed *I-f* control method applied to an SynRM.

The overall control block diagram of the *I-f* control method proposed in this article applied to SynRM startup is shown in Figure 9. Due to the small damping coefficient of the motor, using a large current amplitude to start the motor will cause significant speed fluctuations. Frequency correction is required to continuously adjust the *θ_err_* between $\omega_{i}$ and $\omega_{e}$ to make the torque as smooth as possible. This method has been widely validated and applied [17,18,19]. The method proposed in this article will use frequency correction throughout the entire process. Due to the sudden change in current in the current-down state, a band-pass filter (BPF) should be used instead of just a high-pass filter (HPF) for response power changes. The specific manifestation is that the current is used to calculate power passes through a low-pass filter (LPF). And the filtered current can be used in the current regulator to prevent unstable output in the current-down state and angle adjustment state.

## 5. Experimental Results

### 5.1. Experimental Setup

**Figure 10:**
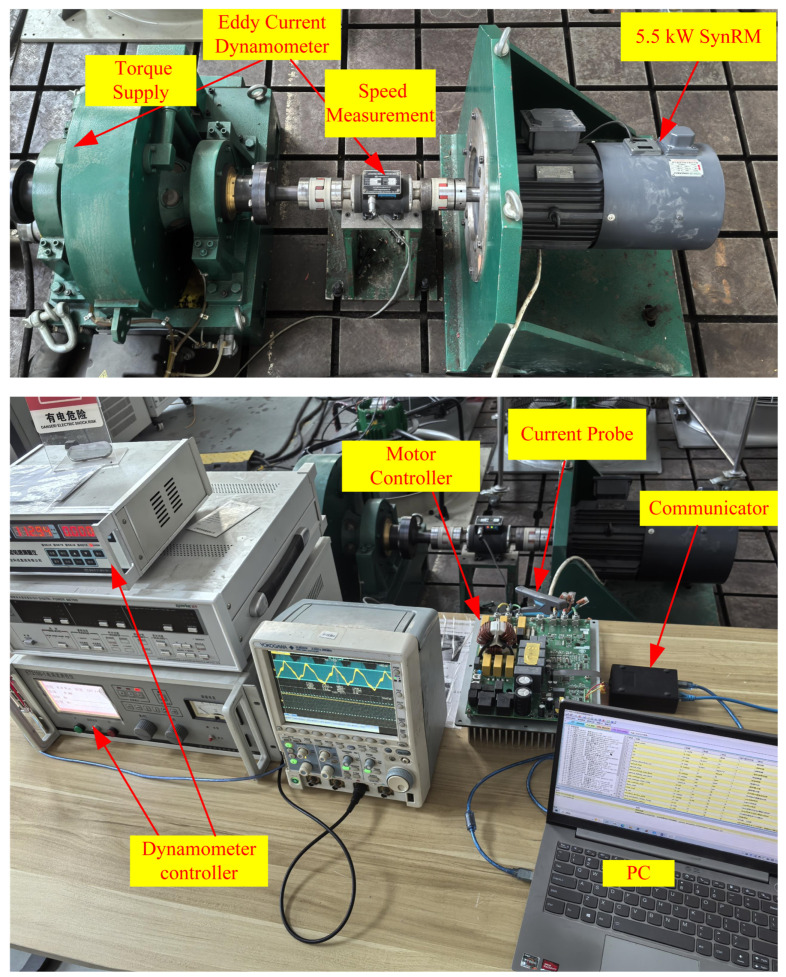
Schematic of the proposed *I-f* control method applied to an SynRM.

The experimental platform used is shown in Figure 10, where an eddy current dynamometer is used to apply load, and the accompanying photoelectric encoder can provide speed and angle information, but these data are only for comparison and will not be used in motor control. A current probe is used to sample the current, a signal oscilloscope is used to record and display current signals, and a PC is used to communicate and control with the motor controller through a communicator. The parameters of the 5.5 kW motor are displayed in Table 1. The controller uses an RXR5F524T8ADFM chip form Renesas. The PWM carrier frequency is 10 kHz, and the control frequency of the proposed *I-f* control method is the same as this frequency. This frequency is also a suitable high frequency that can run on an MCU based on the complexity of the algorithm. The experiment will be divided into three parts to demonstrate. Firstly, the improvement in the control effect brought about by the proposed current regulator and frequency correction required for the entire experiment will be demonstrated. Secondly, the experimental results of applying the proposed simple inductance calibration method to the motor will be presented. Thirdly, the experimental effects of implementing the current and motor speed under the proposed *I-f* control method according to Figure 7 will be demonstrated. Finally, the performance of the important parameters of using voltage injection to reduce current amplitude and adjust rotor angle in the experiment will be displayed.

### 5.2. The Improvement in Experimental Results using the Proposed Current Regulator and Frequency Correction Function

**Figure 11:**
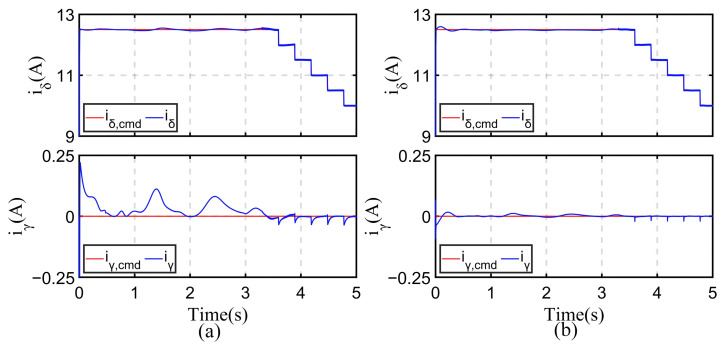
The current regulation effect using different regulators. (**a**) Traditional PI regulator. (**b**) Proposed current regulator.

*I-f* control is a method of controlling motor current, so the accuracy of current control will directly affect the rationality of method use. The current regulator proposed in Section 4.4 can ensure that the current can respond quickly and converge accurately. The experimental images shown in Figure 11 can be obtained by conducting experiments on the proposed method using both the traditional PI and proposed regulator within the first five seconds when the motor is under no-load conditions.

From the figure, it can be seen that there is not much difference in the current control effect for the *δ*-axis, but for the *γ*-axis, traditional PI regulators cannot achieve good current control. Especially at the beginning of control, due to the rapid increase in the current on the *δ*-axis from 0 to $I_{max}$, the axial voltage is rapidly increased using the PI regulator. However, this voltage affects the current on the *γ*-axis due to coupling effects. So, at the beginning, it can be seen that even though the *γ*-axis current is being regulated by the PI regulator, there will still be significant deviation, which is nearly 0.25 A. Afterward, there are multiple current oscillations, which also shows that its response speed is too slow. There are two main impacts. Firstly, there is a large current deviation, indicating poor control stability. Secondly, the method used requires the collection of induced current. If the regulator does not make the controlled current converge quickly, it will cause difficulties in filtering effectiveness and angle judgment.

**Figure 12:**
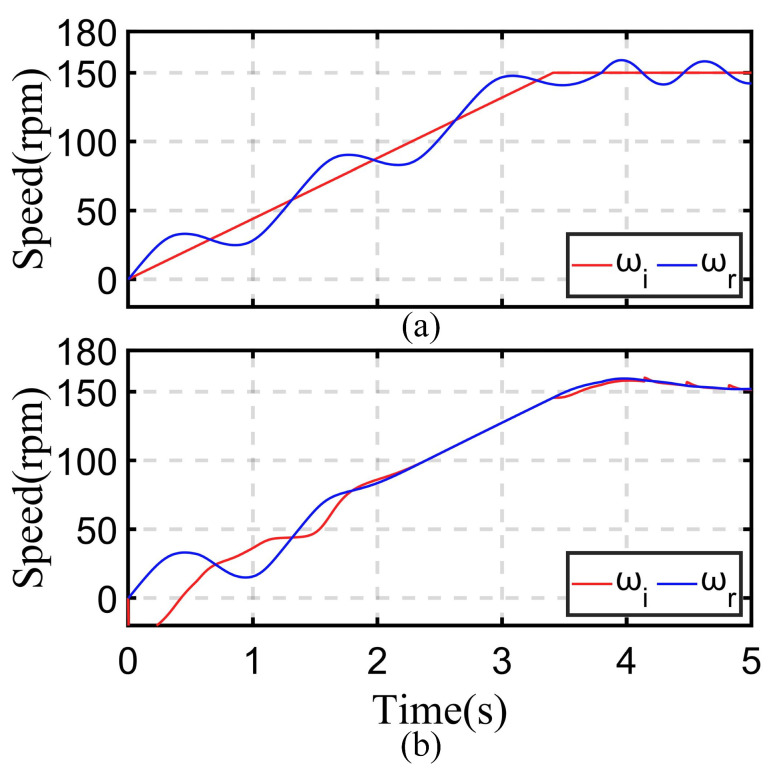
Comparative experiment on motor speed and current frequency results of using frequency correction function. (**a**) Not used. (**b**) Used.

For the use of frequency correction function, this will result in a high degree of coincidence between the current speed and the motor speed, thereby avoiding the problem of difficult angle detection due to motor speed oscillation in subsequent angle detection. A comparative experiment was conducted when the motor was operated under no-load conditions using the *I-f* control method proposed in this paper. The results are shown in Figure 12.

From Figure 12a, it can be seen that when the frequency correction function is not used, the motor speed will experience significant oscillations. The difference between motor speed and current frequency can cause angle difference *θ_err_* to oscillate. This kind of oscillation will seriously affect the detection of motor angle, and when the angle difference $\theta_{err}=\pi /4$, there is a high probability that the *θ_err_* will further decrease, which will cause the motor operation condition to enter the asynchronous region, which is shown in Figure 2. This will cause the motor to fail to start.

In Figure 12b, it can be seen that due to the use of the frequency correction function, the frequency change in the current is quickly adjusted, thus perfectly synchronizing with the motor speed. During the current-down state and angle adjustment state, a stable motor speed can ensure better execution of the algorithm.

### 5.3. The SynRM Inductance Identification Experiment Results

A simple inductance identification method is proposed in Section 4.1, and a subsequent *I-f* control method is proposed based on this method. This method was used to calibrate the inductance of the SynRM used in the experiment. As described earlier, if the entire testing process of the motor is carried out under no-load conditions, when injecting current along a certain axis, for an SynRM, its *d*-axis direction will eventually coincide with the direction of the injected current. For the parameters of the motor, its rated current is $I_{max}=12.5A$. In this experiment, the step size for reducing the current was set to 0.5 A, so $I_{switch}$ is expressed as $I_{switch}=\frac{nI_{max}}{25\sqrt{2}}\;\left(n=1,2\ldots 25\right)$. In each current amplitude step, the current switched between rising and falling 200 times, and the current boundary was set to 0.6 A, which is $I_{border}=0.3A$.

**Figure 13:**
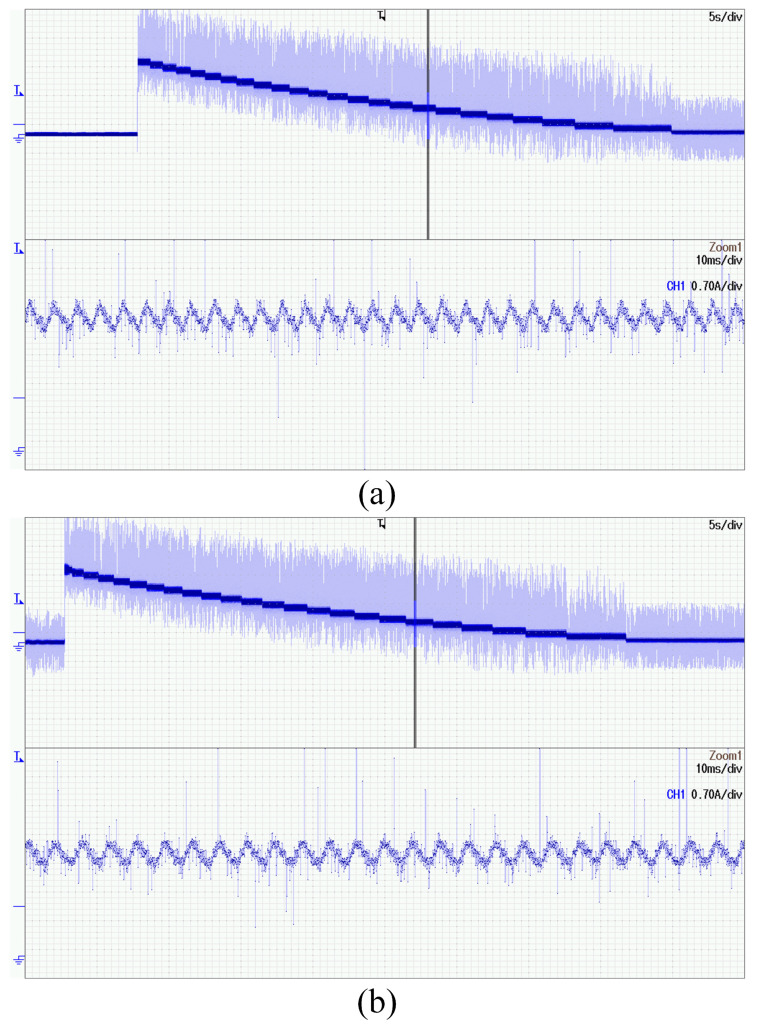
Phase-A current image of inductance identification experiment. (**a**) *L_d_* identification. (**b**) *L_q_* identification.

When measuring the *d*-axis inductance, a certain amplitude of current was first injected into phase-A of the motor to complete the pre-positioning, and then a voltage was injected into phase-A to calibrate the inductance value. The injected voltage amplitude used was $u_{inj,d}=30V$. The results of collecting phase-A current are shown in Figure 13a. For measuring *q*-axis inductance, a certain amplitude current was injected into the π/4 rad electrical angle position of the lagging motor phase-A to complete the pre-positioning. Then, a voltage was injected into phase-A to calibrate the inductance value, with an injection voltage amplitude of $u_{inj,q}=20V$. The results of collecting phase-A current are shown in Figure 13b. The time axis in the figure is 5 s/div, and the current amplitude axis is 3.5 A/div. Both current images are accompanied by a zoom-in to display the specific changes in current under high-frequency voltage injection. The *d-q* axis inductance calculated using the set coefficients combined with the image for each current magnitude is shown in Table 2. In the table, n is used to represent the magnitude of the current $I_{switch}$, combined with its expression.

To demonstrate the accuracy of inductance identification, the motor model identification method proposed in [26] was used to identify the same motor model. The identified parameter results are shown in Table 3. The drawing results of the axial inductance of the *d-q* axis itself are shown in Figure 5. The simple inductance identification method proposed in this article has a disadvantage in accuracy compared to complex identification methods. However, in comparing Figure 5 and Table 3, for the experimental environment in this paper, the accuracy is sufficient for the proposed *I-f* control method.

### 5.4. Overall Performance of the Proposed I-f Control Method

In order to demonstrate that the proposed method can effectively achieve fast startup of SynRMs, the motor was tested under three different operating conditions: no-load condition, half-rated-load condition, and rated-load condition. According to the theory of the identification inductance method mentioned above, the rotor of the motor can be pre-positioned by simply injecting current. In all experiments, 8 A current was injected into phase-A first to position the *d*-axis of the motor in the phase-A direction. Afterward, the motor was accelerated to 150 rpm by gradually increasing the frequency of the current at a ratio of 3π Hz/s with a current amplitude of 12.5 A. The frequency correction function always operated during the acceleration process, using a proportional coefficient of $k_{\omega }=0.15$. The coefficient $w_{c}$ in the current regulator was 40 rad/s. The control frequency and carrier frequency were both 10 kHz.

Unlike in PMSMs, reluctance torque is all electromagnetic torque in SynRMs, which makes their startup time extremely long. For starting the motor directly using the high-frequency voltage injection method with the same motor as in this article, it takes about 20 s. The experimental results are shown in [28].

**Figure 14:**
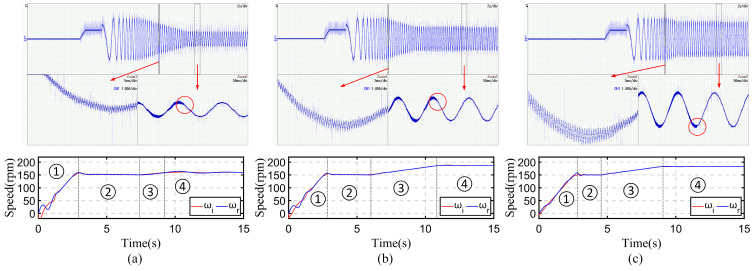
Experimental results of the proposed *I-f* control method for A-phase current and motor speed images under three different load conditions. (**a**) No-load condition. (**b**) Half-rated-load condition. (**c**) Rated-load condition.

When the proposed *I-f* method is successfully implemented, the current and speed of the motor will smoothly transition in four stages as shown in Figure 7. The A-phase current and motor speed of the motor are recorded under three different load conditions, and the rotation speed of the current is also recorded for comparison. The results are shown in Figure 14.

For the A-phase current image, the time axis is 2 s/div, and the current axis is 5 A/div, and two zoom-ins are used for a more detailed display, corresponding two zoomed-in images as indicated by the arrows. The position of Zoom1 is in the current-down state of the control method; Zoom1 displays the amplified phase current to show the induced high-frequency current component caused by the injection voltage. The position of Zoom2 is at the switch moment between the angle adjustment state and the sensorless control state; Zoom2 displays the timing of high-frequency voltage injection cancelation and shows the steady current of sensorless control. At the position marked in the red circle, it can be clearly seen that the induced current caused by the injected voltage has disappeared, and at this moment, it enters the sensorless control state. At this moment, the input and output of the current regulator, which are the current and voltage vector of the motor, will be inherited on the *d-q* axis through a coordinate transformation of $\theta_{err}=\pi /4$, as the initial state for subsequent sensorless control.

For the speed image, the current rotation speed and motor speed are displayed. From the figure, it can be seen that due to the frequency correction function, there is no significant fluctuation in the motor speed during the entire speed-up state. In the current-down state, due to the decrease in current amplitude, the power will decrease, which will gradually reduce *θ_err_*. Frequency correction will cause some protrusions in the current rotation speed image, which is the phenomenon of constantly adjusting the angle. Afterward, the motor speed completely follows the current rotation speed. The switching moments of different states are marked with dashed lines, and each state is labeled with ①, ②, ③, and ④. These identify the speed-up state, current-down state, angle adjustment state, and sensorless control state separately.

Under three different load conditions, the motor speed reached 150 rpm within the speed-up state after approximately 3.3 s. Continuously reducing the current amplitude in the current-down state and continuously increasing the speed in the angle adjustment state to finetune the angle difference *θ_err_* ultimately achieves $\theta_{err}=\pi /4$; at this point, we can consider the motor to be in a highly efficient operating state. Adjusting the angle *θ_err_* to π/4 means the end of *I-f* control, at which moment the control method switches to a closed-loop sensorless control method. When the motor is under the no-load condition, the *I-f* control takes about 8.5 s, the current amplitude finally reaches 4.5 A, and the motor speed reaches 158 rpm. When the motor is under the half-rated-load condition, the *I-f* control takes about 11.5 s, the current amplitude eventually reaches 6.5 A, and the motor speed reaches 189 rpm. When the motor is under the rated-load condition, the *I-f* control takes about 8.3 s, the current amplitude is finally 10.5 A, and the motor speed reaches 181 rpm. The overall phenomenon conforms to the expected current and speed in Figure 7, indicating that the proposed *I-f* method is effective.

### 5.5. Experimental Results of Reducing Current Amplitude and Adjusting Angle Difference θ_err_ Process

**Figure 15:**
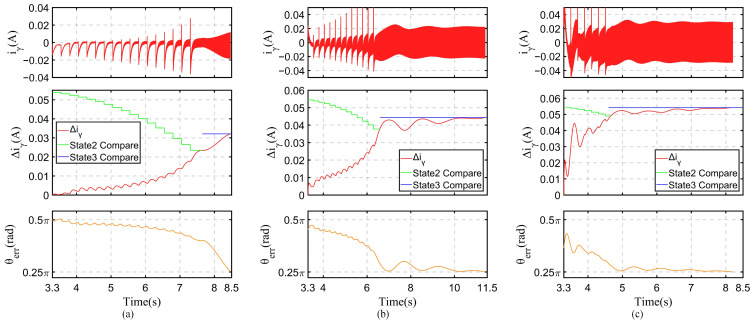
Display of *i_γ_*, Δ*i_γ_*, and *θ_err_* during the current-down state and angle adjustment state. (**a**) No-load condition. (**b**) Half-rated-load condition. (**c**) Rated-load condition.

The main challenges of the *I-f* control methods are improving efficiency and smoothly switching to the sensorless control method. Improving efficiency requires reducing the current after reaching the command speed, and smoothly switching requires determining the position of the rotor. The method proposed in this article can solve the problem of existing methods being unusable for current amplitude and angle control on SynRMs. The control of current and angle occurs in the second and third stages, which are the current-down state and angle adjustment state. According to Section 4.2, if the square-wave-type voltage represented by (17) is injected into the *δ*-axis, it can be inferred from (19) that Δ*i_γ_* can be directly used to calculate *θ_err_*. Due to the control frequency being 10 kHz, if the direction of injected voltage is switched every control cycle, Δ*i_γ_* will be very small, which can easily cause sampling errors and angle judgment errors. The direction of voltage injection should be switched every four control cycles; that is, set *T* = 0.0004 s in (18). This means that the injection voltage frequency is 1250 Hz, and *V* = 30 V is selected as the injection voltage amplitude. The proposed *I-f* control method was applied to an SynRM under the three different load conditions, recording *i_γ_*, Δ*i_γ_*, and *θ_err_* in these two stages. The results are shown in Figure 15.

During the current-down state, the amplitude of the current decreases. As analyzed in Section 3.1, the angle difference *θ_err_* gradually decreases, and when combined with the frequency correction function, the change in *θ_err_* causes significant fluctuations in *i_γ_*, as shown in the current image in the first row of Figure 15. In recording the value of *i_γ_* at each moment the voltage injection direction is switched, after taking the absolute value of the difference between two adjacent recorded values, the red curve in the second row of images can be obtained. The green line is $\frac{{\left(n-1\right)}^{2}\left(L_{d}-L_{q}\right)VT}{2n^{2}L_{d}L_{q}}$, and the blue line is $\frac{\left(L_{d}-L_{q}\right)VT}{2L_{d}L_{q}}$. From the figure, it can be seen that as the current amplitude gradually decreases, *θ_err_* gradually increases, and Δ*i_γ_* also gradually increases. Based on the description in Section 4.3, it can be inferred that the green and blue lines represent the comparative numerical curves of the current-down state and angle adjustment state, respectively. When Δ*i_γ_* and $\frac{{\left(n-1\right)}^{2}\left(L_{d}-L_{q}\right)VT}{2n^{2}L_{d}L_{q}}$ are equal, switch to the angle adjustment state. When Δ*i_γ_* and $\frac{\left(L_{d}-L_{q}\right)VT}{2L_{d}L_{q}}$ are equal, switch to sensorless control. From the figure, it can be seen that for the three different load conditions, the switching times are 7.6 s, 6.5 s, and 4.6 s, respectively.

When the control is in the angle adjustment state, the value used for comparison will become the expression shown in (19), represented by a blue line in the figure. When Δ*i_γ_* is equal to it, switch to sensorless control. The sensorless control method used is the one proposed in [29], and the control coefficient is specifically set to demonstrate better experimental results. Since it is not the focus of this article, further description will not be provided.

In these two stages, an image of the angle difference *θ_err_* is displayed in the last row. From the graph, it can be seen that as the current amplitude decreases, *θ_err_* gradually decreases. When *θ_err_* rapidly decreases, it is immediately switched to the angle adjustment state. Then, in slowly increasing the speed and adding some damping torque, $\theta_{err}=\pi /4$ is achieved.

## 6. Conclusions

The current frequency (*I-f*) control method has been widely applied and studied as a method for quickly starting motors. However, existing research has mainly focused on permanent magnet synchronous motors (PMSMs), and applying existing studies to synchronous reluctance motors (SynRMs) may result in the inability to control the current amplitude and rotor angle. The design of current regulators also presents certain difficulties, and SynRMs exhibit flux saturation phenomenon. This article provides a detailed analysis of the reasons for these issues and, based on the above issues, proposes a simple inductance identification method and further proposes a four-stage *I-f* control method. With the use of voltage injection to assist in determining the angle of the motor, and in reducing the current and increasing the command speed, the efficiency is ultimately improved and the angle is determined. Thus, it is possible to successfully switch to sensorless control. The frequency correction function is used throughout the entire method to eliminate speed fluctuations, and the current regulator proposed in this article can ensure the success of the experiment. Finally, the experimental results demonstrate the effectiveness of this method.

## Figures and Tables

**Table 1:** Parameters of the 5.5 kW SynRM.

Parameter	Value
Pole Pairs	2
Resistance	0.25 Ω
Rated Current	12.5 A
Rated Voltage	380 V
Inertia	0.108 kg·m^2^

**Table 2:** Identification results of *d-q* axis inductance.

n	1	2	3	4	5	6	7	8	9	10	11	12
*L_d_* (mH)	44.01	43.84	43.68	43.40	43.05	42.62	42.12	41.57	40.96	40.29	39.59	38.84
*L_q_* (mH)	20.35	19.92	19.30	18.03	16.72	15.46	14.31	13.30	12.42	11.66	11.01	10.46
n	13	14	15	16	17	18	19	20	21	22	23	24
*L_d_* (mH)	38.07	37.26	36.44	35.60	34.75	33.89	33.03	32.18	31.32	30.48	29.65	28.83
*L_q_* (mH)	9.982	9.576	9.288	8.927	8.667	8.440	8.242	8.067	7.914	7.778	7.657	7.550

**Table 3:** Identification results of the SynRM using the complex identification method.

*A_d_*	*B_d_*	*C_d_*	*A_q_*	*B_q_*	*C_q_*	*K_d_*	*K_q_*	*D_dq_*
0.4812	0.0882	0.0016	0.0401	0.3799	0.0062	576	576	−4.8122

## Data Availability

The data presented in this study are available on request from the author Yibo Guo (email: guoyb22@jlu.edu.cn). The data are not publicly available due to privacy.
